# Linking data on women in public family law court proceedings concerning their children to mental health service records in South London

**DOI:** 10.23889/ijpds.v6i1.1385

**Published:** 2021-02-24

**Authors:** RJ Pearson, A Jewell, L Wijlaars, S Bedston, E Finch, K Broadhurst, J Downs, R Gilbert

**Affiliations:** Population, Policy and Practice Research and Teaching Department, UCL Great Ormond Street Institute of Child Health, London, UK; NIHR South London and Maudsley NHS Foundation Trust Biomedical Research Centre, London, UK; Centre for Child and Family Justice Research, Lancaster University, Lancaster, UK; Central Acute and Addictions Directorate, South London and Maudsley NHS Foundation Trust, London, UK; Institute of Psychiatry, Psychology and Neuroscience, Kings College London, London, UK † indicates joint senior authorship

## Abstract

**Introduction:**

Maternal mental health problems and substance misuse are key risk factors for child neglect or abuse and court-mandated placement into care. Linkage between mental health records and family court data could raise awareness about parent mental health needs and inform approaches to address them.

**Objectives:**

To evaluate data linkage between administrative family court data and electronic mental health records for a population-based mental health service for 1.3 million people in South London.

**Methods:**

We deterministically linked administrative family court data for women (n=5463) involved in care proceedings in South London with service user records from the South London and Maudsley NHS Mental Health Trust (SLaM). We restricted the cohort to women involved in proceedings between 2007 and 2019, in local authorities where SLaM solely provides secondary/tertiary mental health services and the Improving Access to Psychological Therapies (IAPT) (n=3226). We analysed the associations between match status and sociodemographic/case characteristics using multivariable logistic regression.

**Results:**

Two-thirds (2317/3226; 66%) of women linked to a SLaM service user record at some point; most (91%) who linked accessed secondary/tertiary mental health services, indicating serious mental illness. Accounting for possible missed matches, we estimated that 70-83% of women accessed SLaM services at some point. Older women at index proceedings (>35yrs OR: 0.69, 95%CI: 0.54-0.88vs <25yrs) and Black women or women from other ethnic groups (Black ethnic groups 0.65, 0.50-0.83; other ethnicity 0.59, 0.43-0.81 vs White ethnic groups) had lower odds of linking. Odds of linking were higher for women with an infant in proceedings (1.42, 1.18-1.71), or with curtailed/terminated parental responsibility (1.44, 1.20-1.73).

**Conclusion:**

Our linkage supports growing evidence of a high burden of mental health problems and substance misuse among women whose children enter care in England, compared to the general population. Research using this linkage should inform strategies to address the considerable mental health needs of vulnerable women and their children

## Key messages

- Two-thirds of women whose children were subject to care proceedings in four South London local authorities had linked mental health records at some point during a 12-year period: 91% of these women had contact with secondary or tertiary services indicating serious mental illness.
- These findings may underestimate need due to poor identifier quality (e.g. missing date of birth) and ascertainment biases relating to unequal access to healthcare. Sensitivity analyses estimated that 70-83% of women may have used mental health and substance misuse services over the study period.
- Our linkage demonstrates the feasibility of establishing cross-sectoral linkages using administrative data sets and electronic patient records in England. Use of these linked data is encouraged and interested researchers should contact the CRIS administrator at cris.administrator@slam.nhs.uk.

## Introduction

While mental health problems (including those related to substance misuse) are very common among mothers in England [1], little is known about the healthcare needs, or maternal characteristics associated with health service use, among women (approx. 16-17,000 each year) whose children are subject to court-mandated entry into care in England [2]. In the absence of robust empirical evidence regarding prevalence of, or the detail of mental health need (specific to this population), services may be limited in their ability to treat mental health and prevent the reception of children into state care. Anecdotal evidence is that some women with mental health problems involved with children’s services and the courts will retain the care of their children – yet others do not. However, in the absence of a differentiated picture of mental health need, it is difficult to address or explain the varied outcomes that result from public family court proceedings (‘care proceedings’) [3].

Furthermore, involvement in care proceedings and having a child placed in care can, itself, have a negative impact on maternal mental health and substance misuse [4–6]. This is turn can lead to further child protection intervention if women subsequently become pregnant. Recent research using family court data has estimated that one in four women involved in an initial set of care proceeding later return to court within seven years [7], many of whom had mental health or substance misuse problems and with the majority triggered by subsequent pregnancy [8]. This figure is likely to be an underestimate of the volume of children separated from parents, as children can also enter care via out-of-court arrangements (often referred to as voluntary accommodation under s.20 of the Children Act 1989), which are not recorded in family court data [9].

Traditional prospective cohort studies and surveys, which typically include only a small sample of the general population, are not well suited for research into the associations between parental health service use and child entry into care as very few (~3.3%) children in England ever enter care [10]. In addition, families at higher risk of child protection are likely hard to engage and retain in research that relies upon self-reported measures [11,12]. To overcome these barriers, researchers in Australia, Canada, the US and Wales, among others, have linked together data from large-scale administrative linked datasets, based on full-service populations - for example, child protection records and healthcare records - to generate large amounts of quantitative evidence on the association between maternal mental health, child neglect and abuse, and child placement in state care [13–16]. In England, similarly linked data resources are urgently needed to identify opportunities for an improved response to maternal mental health need through the family court, children’s social care, and health services [17,18].

To address this, we created a linked data resource, combining mental health and substance misuse service user records with records for women involved in care proceedings in eight local authorities in South London served by the South London and Maudsley NHS Mental Health Trust (SLaM). In collaboration with the Maudsley Biomedical Research Centre (BRC), this linkage has been established as a research database to be used in conjunction with data on the broader population of approximately 200,000 women accessing SLaM’s services. Together, these data will be used to generate a wealth of evidence on the interrelationship between women’s mental health and substance misuse and their involvement in care proceedings in England and will draw comparison with the broader population of women using SLaM’s services. This report describes how we linked data on care proceedings via deterministic rules to SLaM’s case register of service users and how we evaluated the quality of this linkage. 

## Methods

### Public Involvement

We consulted the Maudsley BRC Data Linkage Service User and Carer Advisory Group with our initial research proposal to establish this research database [19]. The group constitutes people with lived experience of mental illness, all of whom have an interest in mental health research involving data linkage. In addition, we also held a focus group with women and practitioners from one of the Pause project’s South London programmes [20]. Pause provide a programme of support to women who have had children removed from their care via the family court and this programme currently operates in 32 English local authorities. Finally, we discussed our research proposal with members of the Addiction Service User Research Group, which is a local group of drug and alcohol service users who meet regularly and provide advice and support to those undertaking studies relating to addiction, organised jointly by the King’s College London Addictions Department and the Aurora project in Lambeth. At each of these events, we discussed our data linkage methods and research strategy, receiving positive feedback.

### Study cohorts

The study cohort comprises public family court records for 5463 unique IDs assigned to women identified as the mother of a child subject to care proceedings that began or ended between April 2007 and March 2019 and were brought by one of eight South London local authorities. The local authorities were Bexley, Bromley, Croydon, Greenwich, Lambeth, Lewisham, Southwark and Wandsworth.

SLaM is the sole provider of secondary and tertiary mental health services only in Croydon, Lambeth, Lewisham and Southwark. Therefore, much of this paper focussed on the sub-cohort of 3226 women involved in care proceedings in these four local authorities. The decision to include women in proceedings in Bexley, Bromley, Greenwich and Wandsworth for linkage was taken to improve sample sizes and generalisability of findings for research focussing on the use of specific mental health services among this population. For example, SLaM was the sole provider of substance misuse services to Greenwich and Bexley between April 2007 and March 2019, and to Wandsworth between August 2015 and March 2019. 

### Data resources

#### Mental health service user records

In England, care for mental health problems and substance misuse, including counselling and antidepressant/anxiolytic prescribing, is typically provided by general practitioners (GPs) in the first instance. GPs and other health or social care practitioners can also refer patients who do not respond to treatment, or who require specialist mental health care that cannot be provided by a GP, to secondary and tertiary mental health services provided by an NHS mental health trust (such as SLaM). In addition, people experiencing anxiety disorders and depression can self-refer, or, again, be referred by a health or social care practitioner, to community-based services providing psychological therapies (such as cognitive behavioural therapy), known as IAPT (Improving Access to Psychological Therapies) services.

SLaM is the sole provider of NHS secondary and tertiary mental health services in its four constituent local authorities (Croydon, Lambeth, Lewisham and Southwark), which, combined, have an estimated population of 1.3 million [21,22]. For much of the study period, SLaM was also the sole provider of substance misuse services to these four local authorities (supplementary appendix, Table A1). SLaM additionally delivers some services, mostly for substance misuse, to four neighbouring local authorities (Bexley, Bromley, Greenwich and Wandsworth) and provides several specialist national services including a 12-bed mother and baby unit.

**Figure 1: The five electronic patient record systems that feed into the CRIS database:**
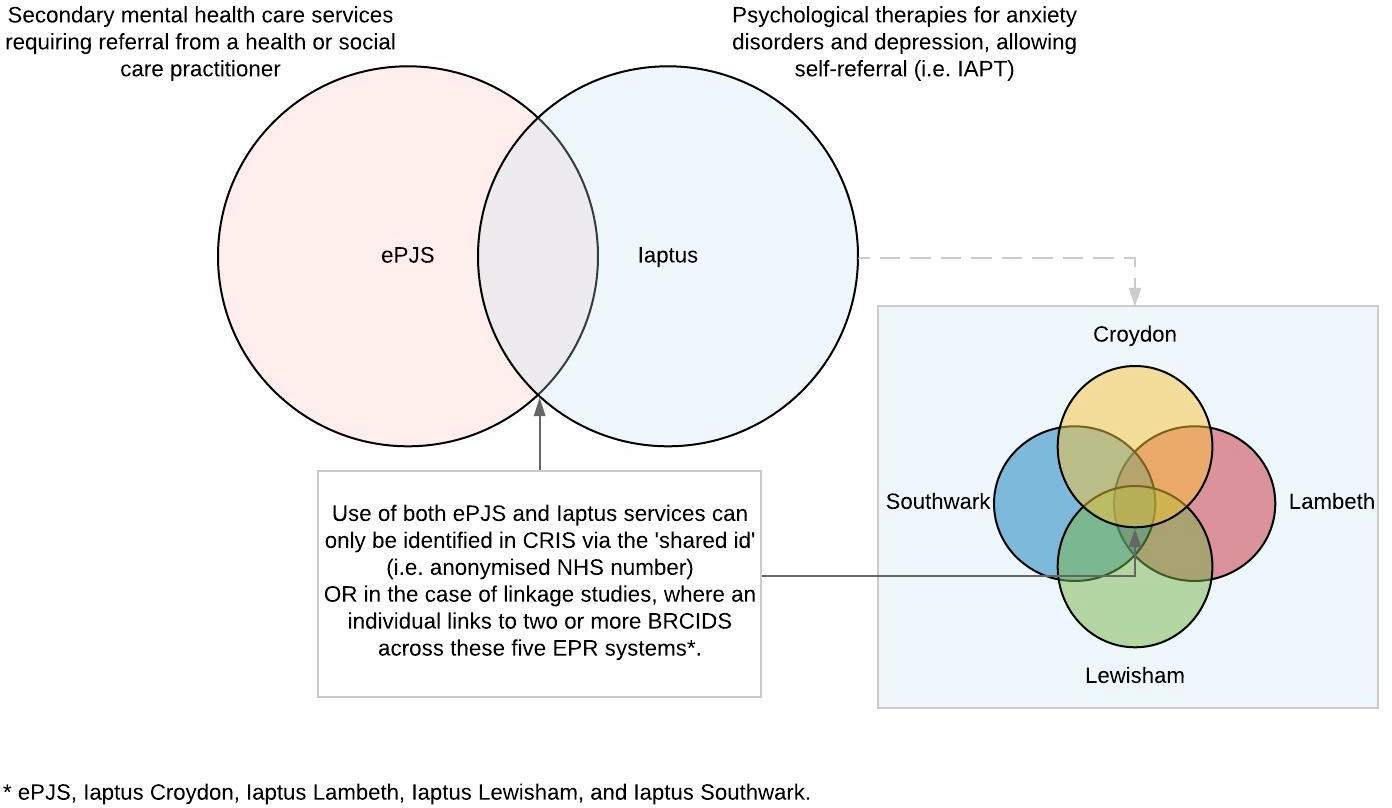


Most of SLaM’s services use a bespoke electronic patient record (EPR) system, the electronic patient journey system (ePJS), to record information on service users. However, SLaM also operates IAPT programmes in Croydon, Lambeth, Lewisham and Southwark, which use the Iaptus EPR system. Therefore, SLaM hosts five EPR systems: ePJS, Iaptus Southwark, Iaptus Croydon, Iaptus Lambeth and Iaptus Lewisham, though service user information is not shared between them (Figure 1).

To facilitate research, SLaM established the Clinical Record Interactive Search (CRIS) database in 2007, which contains anonymised records from each of these five EPR systems (Figure 1) [22,23]. In CRIS, each mental health service user, within each of the EPR systems, is assigned a pseudonymised unique identifier (the ‘BRCID’), allowing researchers to link an individual’s data longitudinally. The laptus and ePJS EPR systems also capture the service user’s NHS number, where available, which is anonymised in CRIS (the ‘shared id’) and can be used to identify individuals using both ePJS and IAPT services, and to link information between these (Figure 1). In addition to structured fields (e.g. dates, diagnoses and medications), CRIS includes anonymised unstructured, free-text fields (such as clinical progress notes, discharge reports and other correspondence) [24]. This enables researchers to utilise the full electronic health record using natural language processing applications or manual review [22].

#### Public family court case records

Where local authorities seek the removal of a child from parents’ care through court order under the Children Act 1989, they must submit an application to the family court [25]. The application is also sent to the Cafcass (Children and Family Court Advisory and Support Service), an organisation which, in tandem with a solicitor, represents the child in care proceedings and advises the court on the best interests of the child [26]. At the outset of every set of care proceedings, a Children’s Guardian is appointed by the court and oversees the case until the conclusion of care proceedings. These specialist social workers work independently of the parents and the local authority and therefore, play a critical role in advising the court on whether court orders are needed, as well as the care plan for the child.

The case management system used by Cafcass captures information on all relevant adults and children involved in a public family law application [2]. This includes demographic information such as age, gender, and ethnicity, as well as case information such as who was involved in the case and key dates. The Cafcass records also contain information on final legal orders made. Possible legal order outcomes for children are multiple, but previous research provides a framework for rationalising this data into typical groups of final legal orders for children [7,8]. These groupings are as follows: remaining or returning home (case dismissed or Order of No Order), placed at home (Family Assistance Order or Supervision Order), placed in out-of-home care (Care Order or Secure Accommodation Order), placed with extended family (Special Guardianship Orders or Child Arrangements Orders (known as Residence Order prior to April 2014)), and placed for adoption (Placement Order or Adoption Order) [9,27]. Children may also receive interim legal orders to place them into out-of-home care while care proceedings are ongoing, though Cafcass do not routinely record these orders [2]. These include Interim Care Orders (i.e. a Care Order that is valid for only a fixed period of time) as well as Emergency Protection Orders, which are used to place children at immediate risk of harm into out-of-home care and lasts for up to eight days.

Each person in the Cafcass data is assigned a pseudonymised unique person identifier (‘Cafcass person ID’). Cafcass also captures information on relationships between individuals involved in a set of proceedings. Therefore, researchers can identify ‘case parents’ and follow them longitudinally, if they return to court. Cafcass data is available from April 2007 and therefore may capture only a portion of an individual’s involvement in care proceedings over their life-course. 

### Linkage methods

#### The linkage algorithm

As the Cafcass and CRIS databases do not share a common unique identifier, such as the NHS number, person identifiers were used for record linkage. These included forenames, surnames, aliases, dates of birth and address postcode history (up to three postcodes in Cafcass and up to five in CRIS).

We linked CRIS and Cafcass person identifiers using deterministic matching rules to deal with differences between identifiers for a single person in each data set that were related to erroneous recording or changes to identifiers over time (e.g. moving house or changing names). All women in our study cohort had non-missing forenames and surnames. We allowed a Cafcass person ID to match to multiple BRCIDs and vice versa. In total there were eight steps in our linkage algorithm, represented in descending order of leniency:

- **Step 1:** Exact or partial match on forename and surname. Exact match on full date of birth, and at least one postcode.
- **Step 2:** Exact match on Soundex code for forename and surname [28]. Exact match on date of birth and at least one postcode.
- **Step 3:** Exact or partial match on forename. Exact match on date of birth, and at least one postcode.
- **Step 4:** Exact or partial match on surname. Exact match on date of birth, and at least one postcode.
- **Step 5:** Exact or partial match on forename and surname. Exact match on at least one postcode.
- **Step 6:** Exact or partial match on forename and surname. Exact match on date of birth.
- **Step 7:** Exact match on Soundex code for forename and surname. Exact match on date of birth.
- **Step 8:** Exact match on the first character of the forename, characters 1-3 of the surname, and on full date of birth.

Forenames and surnames in Cafcass and CRIS were split in two (e.g. Forename = ‘Mary-Jane’ would become Forename 1 = ‘Mary’ and Forename 2 = ‘Jane’). Each of the split names were compared pairwise for all four pair combinations. In addition, the unsplit forenames and surnames were compared to any recorded aliases in each data source.

#### Employing the separation principle

**Figure 2: Cafcass-to-CRIS data linkage: applying the separation principle.**
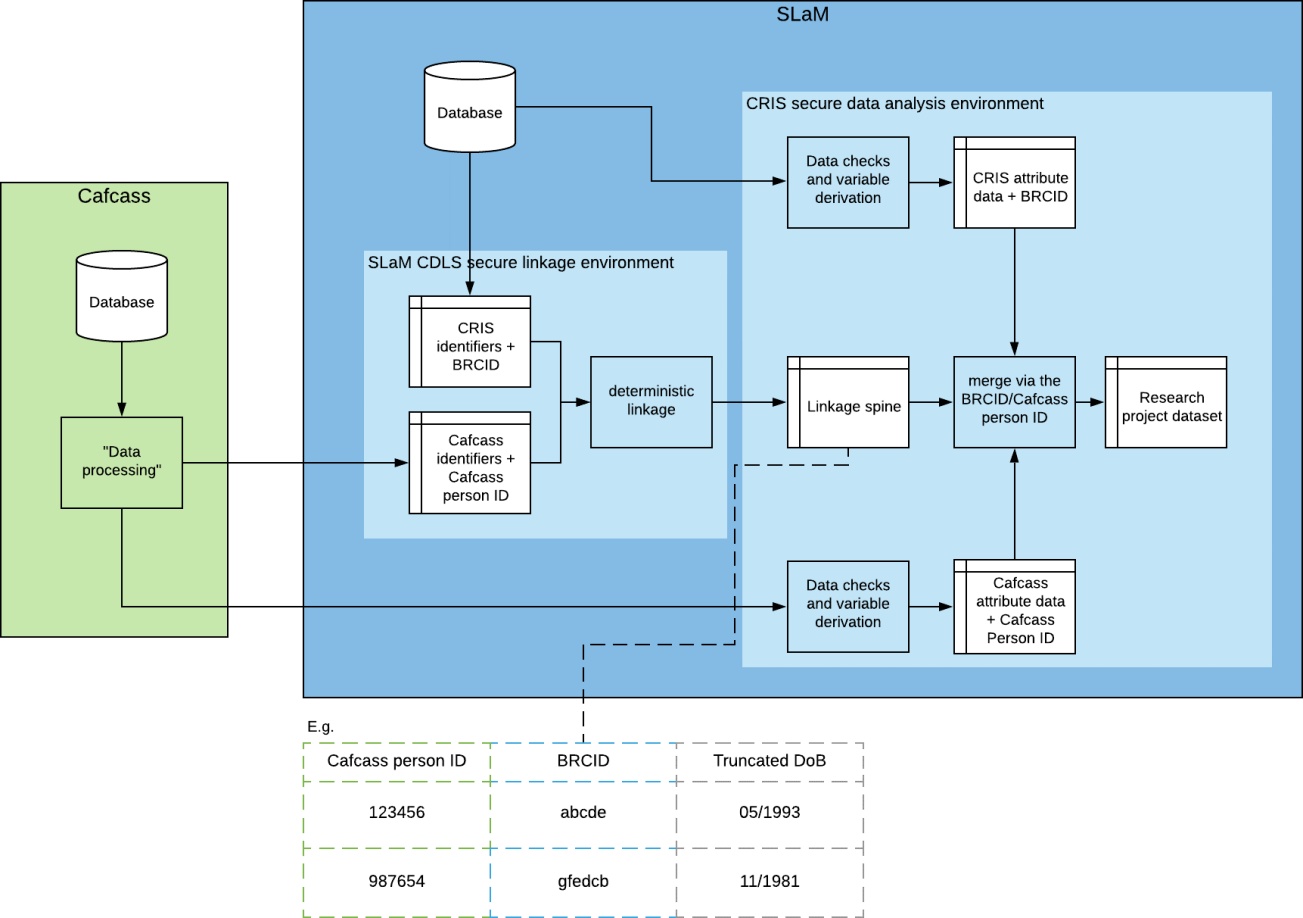


SLaM’s Clinical Data Linkage Service (CDLS) acts as a Trusted Third Party that receives identifiers from data providers external to SLAM and carries out the linkage within the SLaM firewall. To avoid any possibility of individual level care proceedings attribute data being combined with their personal identifier data (i.e. name, date of birth etc), we held person identifiers on a separate database (Figure 2). This is known as the separation principle [22,29]. For all eight steps of linkage, CRIS person identifiers were merged onto the 5643 sets of Cafcass person identifiers. Linkage was carried out with replacement (i.e. many-to-many matches were allowed within and across the eight linkage steps). Once the linkage was finalised, the CDLS removed all person identifier records that did not link. The linkage spine was then created by removing all remaining CRIS and Cafcass person identifier variables (except for date of birth, which was truncated to month and year), leaving only Cafcass person IDs and their corresponding BRCID(s). The linkage spine was then transferred into the CRIS data analysis environment where CRIS and Cafcass attribute data (i.e. mental health/substance misuse service records and Cafcass case information) were merged via the pseudo IDs (i.e. Cafcass person IDs and BRCIDS, respectively) to form the CRIS-Cafcass research data set. 

### Deterministic linkage evaluation

#### Quality assurance for one-to-many matches and lenient linkage algorithm steps

After linkage was completed, we performed a manual review using the pseudonymised linked data set in the CRIS secure data analysis environment (Figure 2). The manual review was carried out for three scenarios including: 1) where a Cafcass person IDlinked to two or more BRCIDs within the same service, 2) where two or more Cafcass person IDs linked to the same BRCID, and 3) where a Cafcass person ID linked to a BRCID via step 8 of the linkage algorithm (due to the leniency of this matching rule). We reviewed anonymised clinical notes, structured fields from SLaM’s child risk screen forms, and Cafcass data on case information and children involved in proceedings. In addition, where Cafcass person IDs matched to a BRCID with an associated shared id (an anonymised NHS number) present, we linked in any additional ePJS and Iaptus BRCIDs associated with the shared id but not already matched to the Cafcass person ID.

#### Estimating ‘true’ and ‘false’ match rates

There are no reference or gold-standard data (i.e. data where match status is known) to compare our linkage results against. Therefore, we could not directly estimate ‘true’ and ‘false’ match rates. After removing false matches and performing de-duplication, we undertook further manual review of de-identified clinician notes and correspondence text for a random sample of 100 BRCIDS that linked. First we searched for mentions of key words related to care proceedings (such as ‘family court’, ‘child protection’ and ‘proceedings’) and checked any positive mentions to ensure they directly related to the patient and fitted into the timeline of proceedings for the linked Cafcass person ID. For women with no positive mentions of these key words, we reviewed clinician case notes and correspondence closest to dates of proceedings. We found evidence to confirm ‘true’ match status for ninety-five women and found insufficient evidence to confirm or contract match status for the remaining five, suggesting few women in the cohort incorrectly matched to a SLaM service user record. 

### Estimating prevalence of mental health service use in South London

We estimated the prevalence of mental health and substance misuse service use among women in the study cohort who were involved in care proceedings in Croydon, Lambeth, Lewisham and Southwark, where SLaM was the sole provider of NHS mental health services. SLaM also provided substance misuse services in these local authorities for much of the study period (supplementary appendix, Table A1). We estimated a plausible range, including a base case, by assuming that a proportion of unlinked records were missed matches. Our formulae were based upon previously reported methods and took into account missingness of date of birth and postcode history in the unlinked records (supplementary appendix, Table A2) [30]. 

### Comparing women in Cafcass who did and did not link to a SLaM service user record

We compared sociodemographic and case characteristics for all remaining women in the study cohort between those who linked to a BRCID and those who did not. Again, we restricted the study cohort to include women who had had at least one set of care proceedings in Croydon, Lambeth, Lewisham or Southwark over the study period.

Using Cafcass attribute data, we produced descriptive statistics for variables that could relate to likelihood of linkage. These variables were derived from all hearings/cases recorded over the study period in Cafcass for the women and included: age at birth of oldest child recorded in Cafcass, age at the beginning of the index (i.e. first recorded over the study period) set of care proceedings, ethnic group (White, Black or Black British, Asian or Asian British, Mixed Heritage, Other, Missing), number of children recorded in Cafcass linked to the mother’s record, age of youngest child involved in proceedings, whether or not the child/ren’s father was ever party to proceedings, Indices of Multiple Deprivation (IMD) 2010 quintiles associated with her address at the index set of proceedings [31], the year (April-March) that their index set of proceedings began, legal orders made in any set of proceedings, and the number of Cafcass records (i.e. sets of proceedings) that women are involved in over the study period.

We used multivariable logistic regression to identify which sociodemographic and case characteristics were informative of match status, with match status as our outcome. Taking the size of the subset cohort into consideration, we decreased the number of categories among some variables to avoid small cell sizes. We also reduced the number of variables included in the analysis to avoid overfitting the model, issues with multicollinearity (i.e. where two or more explanatory variables are strongly correlated with one another), and violating the assumptions for generalised linear models [32].

For example, rather than include all final legal order types, we derived a variable to indicate whether women were ever involved in proceedings that concluded with their parental responsibility being curtailed or terminated (a binary variable where 1 = Care Order, Special Guardianship Order, Child Arrangements Order, Placement Order or Adoption Order, 0 = any other legal order or no legal order. We excluded two variables, before modelling, that we did not consider to be proximally associated with match status - number of children in the Cafcass case management system and father party status in any set of care proceedings - to preserve model parsimony.

We lacked complete information on care proceedings in England that occurred prior to April 2007. To reduce the effect of misclassifying index cases of care proceedings, we conducted a sensitivity analysis and re-ran our model including only women whose index set of proceedings began after March 2010, thereby building in a three-year lookback period to check for prior involvement in care proceedings.

## Results

**Figure 3: Study cohort selection:**
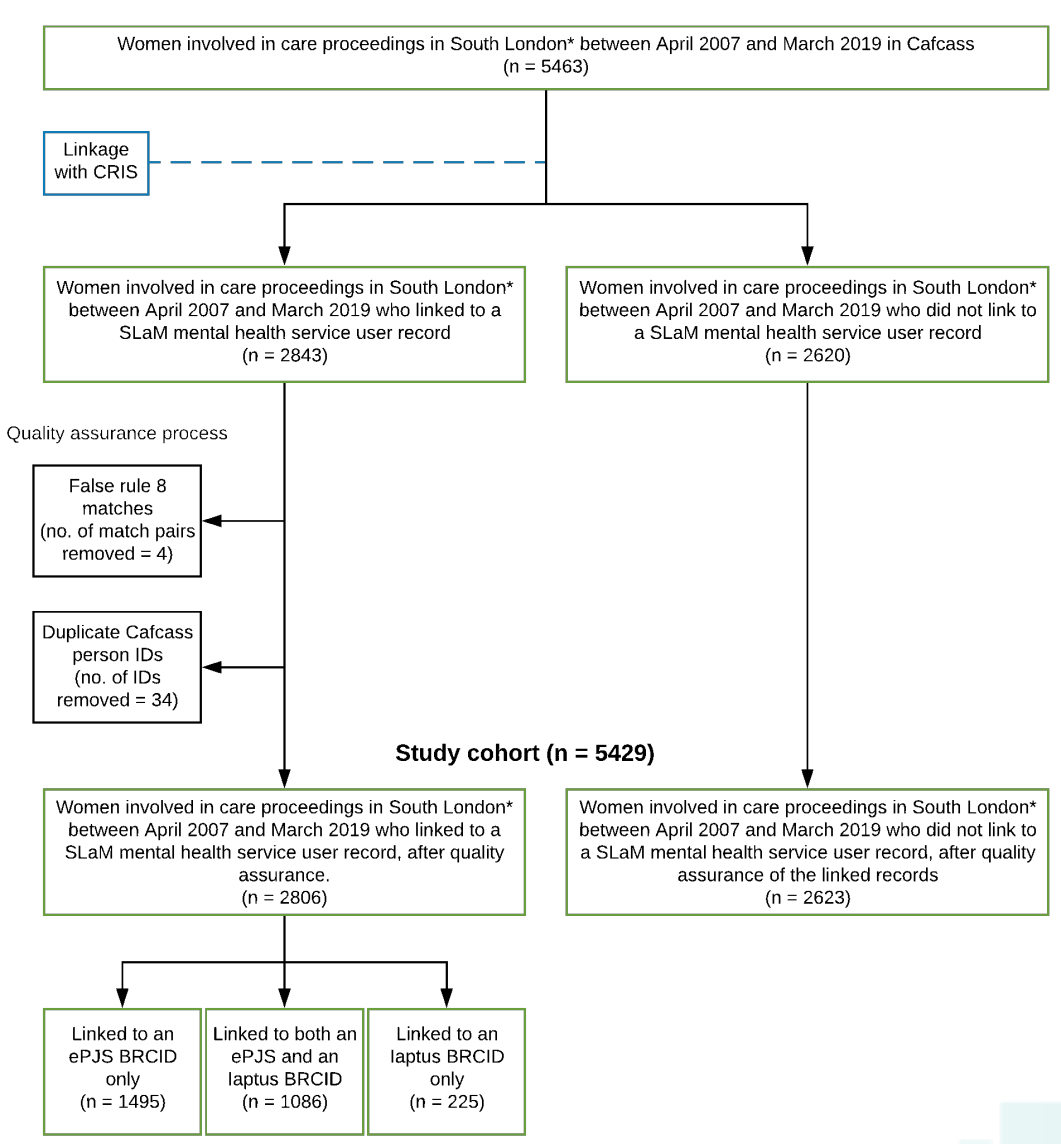
Note: a single cafcass person id may match to multiple BRCIDs. Therefore the removal of match may not result in a cafcass person id no longer being linked to any SLaM mental health service user record.

* Bexley, Bromley, Croydon, Greenwich, Greenwich, Lambeth, Lewisham, Southwark and Wandsworth local authorities.

### Linkage results

More than 50% of women involved in care proceedings in eight South London local authorities between April 2007 and March 2019 linked to a SLaM service user record (Figure 3). We identified and removed four false match pairs from the linkage results; as the Cafcass person ID in one of these false match pairs linked to multiple BRCIDs, removal of these four false match pairs only resulted in three ‘linked’ Cafcass person IDs being reclassified as ‘unlinked’. De-duplication resulted in thirty-four fewer Cafcass person IDs in the study cohort. The number of Cafcass Person IDs that linked to a BRCID at each match step is available in Table A3 of the supplementary appendix. 

### Estimating the prevalence of mental health and substance misuse service use among women involved in care proceedings within local authorities where SLaM is the sole NHS mental health service provider

Among the Cafcass study cohort, 3226 (59.4%) women were involved in Croydon, Lambeth, Lewisham, and Southwark between April 2007 and March 2019, of which 2137 (66.2%) linked (Table 1). Of these, 91.1% accessed SLaM secondary or tertiary care mental health and substance misuse services (i.e. ePJS services). Only 8.9% accessed the IAPT programme alone, which are targeted to individuals experiencing anxiety disorders and depression, indicating a high burden of more serious mental health problems and substance misuse among the women who linked.

**Table 1: Match rate and estimated prevalence of SLaM mental health or substance misuse service user among women involved in care proceedings in Croydon, Lambeth, Lewisham and Southwark between April 2007 and March 2019 (n = 3226):** * Women who were involved in two or more sets of care proceedings in different local authorities will be double counted across the local authority specific figures.

			Missingness among key identifiers in Cafcass for unlinked (%)		Estimated prevalence (%) (plausible range)		
Local authority	Number that link to a BRCID*	Match rate (%)	Missing date of birth only	Missing date of birth AND no valid postcode	Lower limit	Base case	Upper limit

Croydon	502	62.21	20.33	9.84	65.99	73.35	80.72
Lambeth	529	68.43	16.39	11.89	71.59	77.78	83.97
Lewisham	567	65.25	27.15	9.27	68.72	75.68	82.65
Southwark	603	71.45	22.41	11.62	74.30	80.05	85.81
Overall	2137	66.24	21.76	10.56	69.62	76.31	83.00

Applying our plausible value formulae, which takes into account the impact of missingness among date of birth and postcode history in Cafcass on linkage, we estimated that the prevalence of SLaM mental health or substance service use among women involved in proceedings in the constituent local authorities between April 2007 and March 2019 ranged from 69.6% to 83.0% (with a base case of 76.3%). The match rate and estimated prevalence figures varied by local authority and were highest in Southwark and lowest in Croydon (Table 1). The match rate increased in all four local authorities over the study period (Figure 4), likely driven, in part, by better recording of dates of birth over the study period (supplementary appendix, Figure A1).

**Figure 4: Trends in the estimated prevalence of SLaM mental health or substance misuse service use among women involved in care proceedings in Croydon, Lambeth, Lewisham and Southwark over time.**
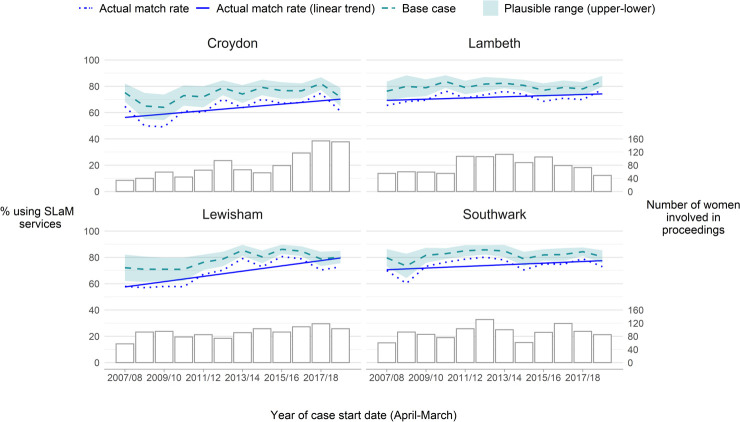


### How did women in Cafcass who linked differ from those who didn’t link?

Almost half of women in the study cohort who were involved in care proceedings in a SLaM constituent local authority (n = 3226) entered parenthood before their 25^th^ birthday and over a quarter were involved in care proceedings before their 25^th^ birthday (Table 2). Date of birth was missing for many (13.7%), with far more women who did not link having no date of birth recorded than women who linked (32.3% vs 4.2%).

Ethnicity in Cafcass was poorly recorded, with almost half of the women missing ethnicity. This missingness varied slightly by match status (44.0% vs 50.5%). Across both groups, most with non-missing ethnicity had White (25.0%) or Black (19.7%) ethnicity recorded. Recording of ethnicity was far better in CRIS; most women with missing ethnicity in Cafcass who linked to a SLaM service user had White (45%) or Black (27%) ethnicity recorded in CRIS (supplementary appendix, Table A7). Furthermore, among those who linked, there was poor agreement (<50%) in the recording of ethnicity between the Cafcass and CRIS data for women whose recorded ethnic group in Cafcass was Asian or Asian British, Mixed Heritage, or ‘other ethnic group’ (supplementary appendix, Table A6).

**Table 2: Characteristics among women involved in proceedings in Croydon, Lambeth, Lewisham and Southwark between April 2007 and March 2019 (n = 3226).** *Other includes the Chinese and ‘other’ categories which captures all other ethnicities. ** Includes unborn children who become subject to an existing set of care proceedings after birth (e.g. the mother is pregnant during proceedings) IMD = Indices of Multiple Deprivation. † Non-mutually exclusive categories ‡ No order includes the following legal outcomes: Application refused, Order of no order, Order refused, Application dismissed, Case by leave withdrawn and Order not made.

Characteristics recorded in Cafcass’ case management system		Non-matches	Matches	Overall
		(n = 1089, 33.8%)	(n = 2137, 66.2%)	(n = 3226)

Age at birth of oldest child recorded in Cafcass’ case management system	Under 20 years	188 (17.3)	616 (28.8)	804 (24.9)
		20-24 years	193 (17.7)	506 (23.7)	699 (21.7)
		25-29 years	168 (15.4)	368 (17.2)	536 (16.6)
		30 years and over	188 (17.3)	558 (26.1)	746 (23.1)
		Unknown	352 (32.3)	89 ( 4.2)	441 (13.7)

Age at index set of proceedings	Under 20 years	64 ( 5.9)	249 (11.7)	313 ( 9.7)
		20-24 years	96 ( 8.8)	361 (16.9)	457 (14.2)
		25-29 years	119 (10.9)	371 (17.4)	490 (15.2)
		30 years and over	458 (42.1)	1067 (49.9)	1525 (47.3)
		Unknown	352 (32.3)	89 ( 4.2)	441 (13.7)

Ethnicity	White or White British	206 (18.9)	599 (28.0)	805 (25.0)
		Black or Black British	226 (20.8)	411 (19.2)	637 (19.7)
		Mixed Heritage	42 ( 3.9)	125 ( 5.8)	167 ( 5.2)
		Asian or Asian British	31 ( 2.8)	30 ( 1.4)	61 ( 1.9)
		Other*	34 ( 3.1)	32 ( 1.5)	66 ( 2.0)
	Missing	550 (50.5)	940 (44.0)	1490 (46.2)

Number of children recorded in Cafcass’ case management system	1	646 (59.3)	1054 (49.3)	1700 (52.7)
		2-3	344 (31.6)	834 (39.0)	1178 (36.5)
		4+	99 ( 9.1)	249 (11.7)	348 (10.8)

Youngest child involved in proceedings		lt;3 weeks old**	150 (13.8)	601 (28.1)	751 (23.3)
		4weeks - 1 year old	224 (20.6)	461 (21.6)	685 (21.2)
		1-4 years old	220 (20.2)	430 (20.1)	650 (20.1)
		5-9 years old	210 (19.3)	359 (16.8)	569 (17.6)
		10-14 years old	216 (19.8)	240 (11.2)	456 (14.1)
		15 years and older	69 ( 6.3)	46 ( 2.2)	115 ( 3.6)

The child/ren’s father is party in at least one set of proceedings?		695 (63.8)	1373 (64.2)	2068 (64.1)

IMD 2010 quintile associated with their recorded address during index set of proceedings	1 – most deprived	364 (33.4)	912 (42.7)	1276 (39.6)
		2	297 (27.3)	686 (32.1)	983 (30.5)
		3	117 (10.7)	230 (10.8)	347 (10.8)
		4	42 ( 3.9)	57 ( 2.7)	99 ( 3.1)
		5 – least deprived	12 ( 1.1)	11 ( 0.5)	23 ( 0.7)
		missing	257 (23.6)	241 (11.3)	498 (15.4)

Year (April-March) that index set of proceedings began	before 2007	44 ( 4.0)	74 ( 3.5)	118 ( 3.7)
		2007/08-2009/10	284 (26.1)	444 (20.8)	728 (22.6)
		2010/11-2012/13	254 (23.3)	571 (26.7)	825 (25.6)
		2013/14-2015/16	231 (21.2)	505 (23.6)	736 (22.8)
		2016/17-2018/19	276 (25.3)	543 (25.4)	819 (25.4)

Final legal orders made in any set of proceedings for at least one child†	Any legal order	936 (86.0)	1935 (90.5)	2871 (89.0)
		No order‡	170 (15.6)	267 (12.5)	437 (13.5)
		Supervision Order or Family Assistance Order	184 (16.9)	486 (22.7)	670 (20.8)
		Care Order	346 (31.8)	658 (30.8)	1004 (31.1)
		Special Guardianship Order/Residence order/Child Arrangements Order (live with)	213 (19.6)	739 (34.6)	952 (29.5)
		Placement Order/Adoption Order	223 (20.5)	517 (24.2)	740 (22.9)

Emergency Protection Order made during any proceedings for at least one child		105 ( 9.6)	159 ( 7.4)	264 ( 8.2)

Two or more records in Cafcass’ case management system		255 (23.4)	807 (37.8)	1062 (32.9)

Most women had only one child recorded in Cafcass over the study period, though women who linked tended to have more children recorded than women who did not link. This is likely linked to the fact that more women who linked had two or more case records (i.e. sets of proceedings) in Cafcass over the study period than women who did not link (37.8% vs 23.4%). Almost half of women had an infant (<1 year old) subject to care proceedings over the study period, again this was higher among women who linked compared to women who did not link (49.7% vs 34.4%). Almost two thirds of women were party to proceedings where both parents had party status. This figure did not differ by match status. At their index proceedings, almost 40% of women had a recorded address within the 20% most deprived LSOAs in England (as per IMD 2010 measures). More women who linked lived in the most deprived areas of England compared to women who did not link (42.7% vs 33.4%). Overall, 15.4% of women had no valid English postcode and missingness among this identifier was higher among women who did not link compared to women who did (23.6 vs 11.3%).

Most women had at least one child with a recorded final legal order in Cafcass. This varied little by match status. There was also little difference between the proportions of women who linked and of women who did not link who had children subject to either a case dismissal or Order of No Order (12.5% vs 15.6%), Supervision Order or Family Assistance Order (22.7% vs 16.9%) , Care Order or Secure Accommodation Order (30.8% vs 31.8%), and Placement or Adoption Order (24.2% vs 20.5%). More women who linked had children subject to Special Guardianship or Child Arrangements Order (34.6% vs 19.6%) than women who did not link. There was little difference between women who did and did not link in the proportion who had a child subject to an Emergency Protection Order during the study period (7.4% vs 9.6%).

#### Identifying sociodemographic and case characteristics that are associated with match status using multivariable logistic regression

**Figure 5: Modelling sociodemographic and case characteristics against match status among women involved in proceedings in Croydon, Lambeth, Lewisham and Southwark between April 2007 and March 2019: odds ratios with 95% confidence intervals (n = 3226):**
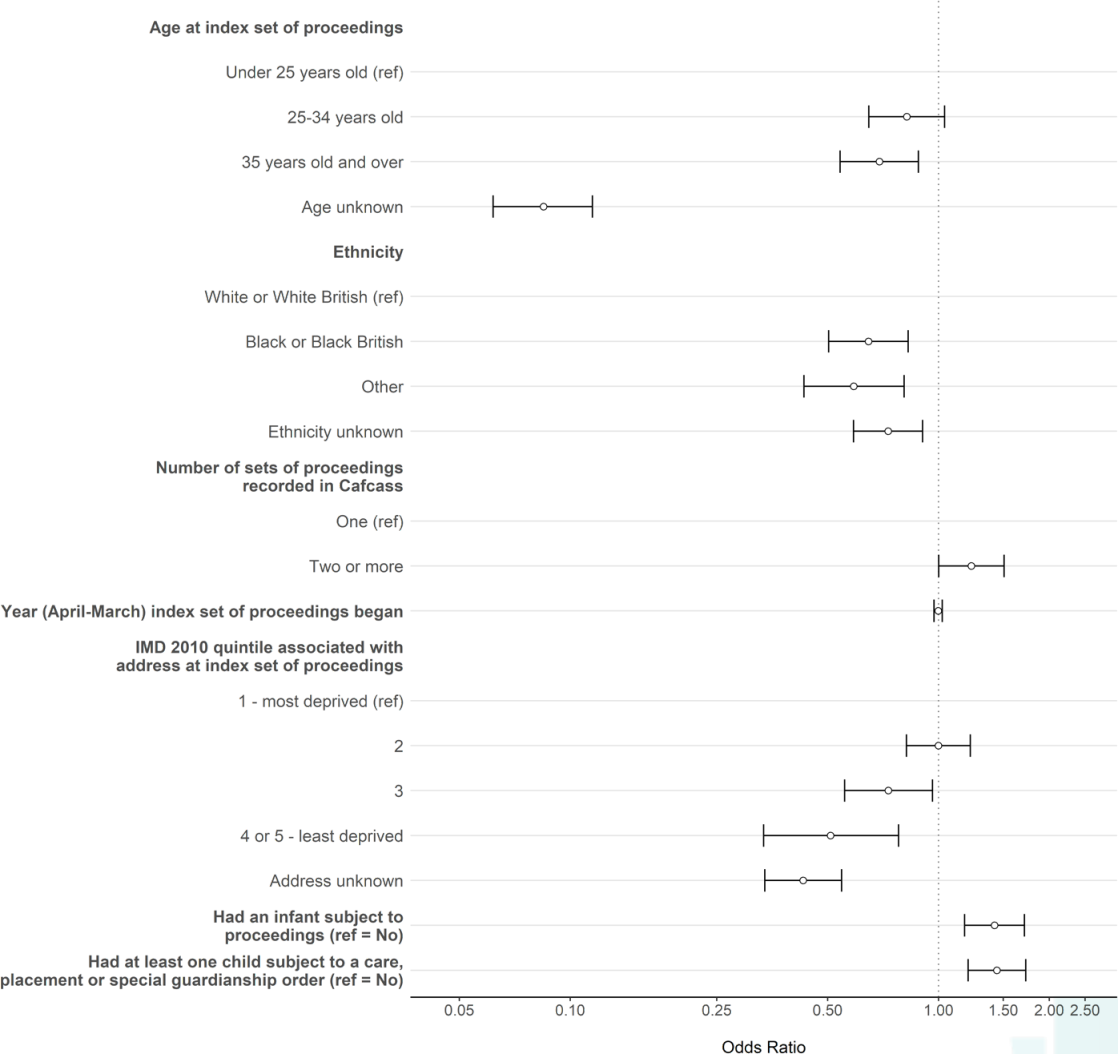


Compared to women who were under 25 years old when their index proceedings began, there was evidence that women who were at least 35 years old (OR: 0.69, 95% CI: 0.54 to 0.88) or whose date of birth was missing in Cafcass (0.08, 0.06 to 0.11) had lower odds of linking to a SLaM service user record (Figure 5). The odds of linking also differed by ethnicity; compared to women recorded as having White ethnicity, there was evidence that women recorded as having Black ethnicity (0.65, 0.50 to 0.83), all other ethnic groups (0.59, 0.43 to 0.81), or with no recorded ethnicity (0.73, 0.59 to 0.91) had lower odds of linking. There was inconclusive evidence of an association between the year that index proceedings began and linking (1.00, 0.97 to 1.02). There was weak evidence that having two or more case records in Cafcass compared to only one (1.23, 1.00 to 1.51) were associated with higher odds of linking. Women who lived in the least deprived areas of England at their index proceedings had lower odds of linking compared to women in the most deprived areas (0.51, 0.34 to 0.78). There was evidence that having an infant subject to proceedings (1.42, 1.18 to 1.71) or a child subject to a Care, Special Guardianship, Residence, Child Arrangements (live with), Placement or Adoption order was associated with higher odds of linkage (1.44, 1.20 to 1.73). These results were consistent with those from our sensitivity analysis (n = 2380). The full results from both the main and sensitivity analyses are available in Tables A4 and A5 of the supplementary appendix.

## Discussion

### Key results

We described successful linkage between administrative data for women involved in care proceedings from Cafcass and electronic mental health and substance misuse service records from the South London and Maudsley NHS Mental Health Trust (SLaM), which serves a population of 1.3 million in South London. Two-thirds of women involved in care proceedings within local authorities where mental health services are solely provided by SLaM (Croydon, Lambeth, Lewisham and Southwark) linked at some point: 91% of these women had contact with secondary or tertiary mental health services indicating serious mental health problems or substance misuse. The prevalence of two-thirds of women in contact with mental health care at some point before November 2019, when linkage was completed, is more than double the previously reported prevalence of one in three women aged 16-54 years old (32.3%) reporting ever being diagnosed with a mental health problem in England in 2014 [33].

Most of the women entered motherhood at a younger age compared to the general population of women giving birth in England [34]. After adjusting for other sociodemographic and case characteristics, women who were younger at their index set of proceedings had higher odds of linking to a SLaM service user record than women who were at least 35 years old, consistent with previous research highlighting higher prevalence of mental health problems among young mothers in the UK [1].

Black women, women from other ethnic groups and women with unknown ethnicity in Cafcass had lower odds of linking to a SLaM service user record than White women. This is likely partly due to systemic barriers to mental health service use that adversely affect Black and minority ethnic communities in South London [35]. There is also evidence that erroneous recording of person identifiers in administrative datasets is more common among Black, Asian and Mixed Heritage ethnic groups compared to White ethnic groups [36,37].

Many women who linked had an infant subject to proceedings and this was associated with higher odds of linking. This association may be driven by strong referral pathways between children’s social services and SLaM perinatal services, which include a 13-bed mother-baby unit, for pregnant women with mental health problems and substance misuse. However, many of these infants subsequently entered care, and rates of infant entry into care are rising in England [38,39]. Therefore, further research is needed to better understand whether improved and earlier perinatal mental health treatment for these women would reduce risk of infant entry into care.

Finally, women whose parental responsibility was curtailed or terminated at the conclusion of a set of proceedings had greater odds of linking and, therefore, may be at greater risk of serious mental illness. Further analyses of the linked data are needed to understand whether the risk of children being subject to these legal orders varies by mental health diagnosis and to characterise women’s SLaM service use before, during and after care proceedings.

#### Strengths and limitations

This is the first linked data resource of its kind in England and our linkage algorithm yielded a high number of matches, of which very few were found to be false (Figure 3). However, due to missingness among date of birth and postcode history in Cafcass, there are likely a number of missed matches leading to underestimation of mental health problems and substance misuse among the cohort. Missingness among these person identifiers in CRIS was minimal (supplementary appendix, Table A8). We also lack information on women whose children enter care under out-of-court arrangements (commonly referred to as voluntary care). Data on ethnicity is poorly recorded in Cafcass, though quality has improved in recent years [2]. Despite finding variation by ethnicity in women’s likelihood to link, we were unable to further categorise the broad ethnic groupings used in our analyses as counts became too small. We did not estimate mental health and substance misuse service use for the four non-constituent local authorities (Bexley, Bromley, Greenwich and Wandsworth) where mental health services are chiefly delivered by other NHS mental health trusts. Nevertheless, the inclusion of Cafcass data for women involved in care proceedings in these neighbouring local authorities where SLaM delivered only some services, such as substance misuse services, may afford researchers who are interested in the use of a particular service among this population group a larger sample size. We identified a small number (2.4%) of duplicated Cafcass person IDs among women who linked but were unable to identify duplicates among unlinked Cafcass person IDs; therefore, the number of women returning to court among the unlinked may be underestimated in these data. However, we expect this to be minimal as Cafcass de-duplicate people upon discovery within their administrative system; we also performed some de-duplication of the Cafcass data prior to linkage for this study (further details on Cafcass data processing can be found in the supplementary appendix). Mental health problems and substance misuse among women in the four local authorities served wholly by SLaM may be underestimated as some women with these conditions may be unknown to services [40], furthermore we lack information from GPs who provide most of the frontline mental health care within the NHS [41]. Finally, Cafcass also captures information on fathers in around two-thirds of cases, which could be linked to CRIS [2]. However, due to the novelty of this linkage and complexity of the ethical permissions required for linkage, we focussed on demonstrating the feasibility and public benefit of linking data on mothers for research.

#### Implications for policy and for future research

Our findings demonstrate the feasibility of linkage between administrative family court data and electronic patient records in England and highlight a high burden of mental health problems and substance misuse among women whose children are subject to care proceedings. Research using these linked data will inform policy strategies within health, family justice and children’s social care to prevent and respond to mental health and substance misuse service needs among women before, during, and after care proceedings. However, further research is needed to identify the most effective ways to improve long-term outcomes for women and their children.

Having demonstrated the feasibility and value of linkage, permissions have now been granted to create a longitudinal cohort of approximately 200,000 women accessing SLaM mental health and substance misuse services between the ages of 16-55yrs old, some of whom were involved in care proceedings between April 2007 and March 2019. With this cohort, we will utilise the whole SLaM record to identify and evaluate healthcare-related risk-factors for involvement in care proceedings, to better understand why some women accessing mental health and substance misuse services are subject to care proceedings while others are not. Findings from these data will inform local service developments to improve the delivery of mental health resources to women living in areas that SLaM serves, as well as national policy and practice across health, family justice, and the wider children’s social care sector. Researchers could expand this linkage by seeking permissions to link Cafcass and mental health records for fathers to examine the interrelationship between maternal mental health, paternal mental health, and care proceedings. 

#### Data Access

The data accessed by CRIS remain within an NHS firewall and governance is provided by a patient-led oversight committee. Subject to these conditions, data access is encouraged and those interested should contact the CRIS administrator at cris.administrator@slam.nhs.uk.

## Acknowledgments

We would like to thank the Children and Family Court Advisory and Support Service for collecting and allowing researchers to access their data. We would also like to thank the entire team at the NIHR Maudsley Biomedical Research Centre Nucleus for enabling access to the CRIS data, for providing ongoing technical support, and training in SQL for CRIS. We are grateful to all who took part in the PPI/E activities; thank you for giving your time so generously and for your input into this linkage and the subsequent analyses. This work uses data provided by service users and collected by Cafcass and the South London and Maudsley NHS Foundation Trust. 

## Contributors

The study was designed by RG, LW, KB, EF and SB. Permissions were sought by RG and LW, with input from AJ, JD and RP. Cafcass data extraction and cleaning was carried out by SB with guidance from LW and RG. CRIS data extraction was carried out by AJ and RP. Data linkage was carried out by AJ, with input from RP and JD. Data wrangling and analysis was undertaken by RP. RP produced the first draft of the manuscript. All authors contributed to revision of the original manuscript and approved the final version.

## Conflicts of interest

The authors declare that they have no competing interests.

## Ethics

We received NHS Research Ethics Committee (REC) approval (ref: 18/SC/0363) and NHS Confidentiality Advisory Group (CAG) approval (ref: 18/CAG/0112) to establish this data linkage without patient consent, as a research database hosted by SLaM. This project was approved by the Cafcass Research Governance Committee and SLaM’s CRIS oversight committee (ref: 19-050). 

## Funding statement

This work was supported by the Nuffield Foundation [grant number KID/42838 to KB, EF, LW, RG, RP and SB]. Funding enabled but did not commission this study from; the National Institute for Health Research (NIHR) Great Ormond Street Hospital Biomedical Research Centre; the NIHR Children and Families Policy Research Unit [to LW and RG]; the NIHR Maudsley Biomedical Research Centre [to AJ], and Health Data Research UK [to RG].

The views expressed are those of the author(s) and not necessarily those of Cafcass, the Nuffield Foundation, the NHS, the NIHR or the Department of Health and Social Care.

## Supplementary Files

Supplementary Appendix

## Abbreviations

BRC = Biomedical Research Centre; Cafcass = Child and Family Advisory and Support Service; CDLS = Clinical Data Linkage Service; CRIS = Clinical Research Interactive Search; ePJS = Electronic Patient Journey System; EPR = Electronic Patient Record; IAPT = Improving Access to Psychological Therapies; IMD = Indices of Multiple Deprivation; SLaM = South London and Maudsley NHS Mental Health Trust.
