# Supplementary material for: Linking data on women in public family law court proceedings concerning their children to mental health service records in South London
Source: Int J Popul Data Sci. 2021 Feb 24;6(1):1385. doi: 10.23889/ijpds.v6i1.1385 (PMC8133060; doi:10.23889/ijpds.v6i1.1385)
Supplement: Supplementary Appendix [file ijpds-06-1385-s001.pdf]

Supplementary Appendix

Table A1: Dates of South London and Maudsley substance misuse service provision to local authorities in London over the study period 2

Table A2: Plausible values for prevalence of mental health service use in Croydon, Lambeth, Lewisham and Southwark 2

Table A3: Number of Cafcass person IDs that link to a BRCID, by match step 3

Table A4: Modelling sociodemographic and case characteristics against match status among women involved in proceedings in Croydon, Lambeth, Lewisham and Southwark between April 2007 and March 2019: odds ratios with 95% confidence intervals (n = 3226) 4

Table A5: Modelling sociodemographic and case characteristics against match status among women involved in proceedings in Croydon, Lambeth, Lewisham and Southwark between April 2010 and March 2019: odds ratios with 95% confidence intervals (n = 2380) 5

Cafcass data processing and cleaning 6

Figure A1: Proportion of Cafcass person IDs who correctly linked to a SLaM service user record, before de-duplication, (n = 2840) who had missing date of birth or no recorded postcode in the Cafcass data 6

Table A6: Agreement in recorded ethnic group between Cafcass and CRIS among Cafcass person IDs that correctly link to a SLaM mental health service user record (n = 2840) 7

Table A7: Ethnic groups recorded in CRIS where ethnic group is missing in Cafcass among Cafcass person IDs that correctly link to a SLaM mental health service user record (n = 2840) 7

Table A8: Missingness among person identifiers in the CRIS database, by electronic patient record system 8

Appendix bibliography 8

Table A1: Dates of South London and Maudsley substance misuse service provision to local authorities in London over the study period

Local authority (London)	Service provision between April 2007 and March 2019
Bexley	Apr 2007 to Mar 2019
Bromley	none
Croydon	Apr 2007 to Aug 2014
Greenwich	Apr 2007 to Mar 2019
Lambeth	Apr 2007 to Mar 2019
Lewisham	Apr 2007 to Dec 2010
Southwark	Apr 2007 to Dec 2015
Wandsworth	Aug 2015 to Mar 2019

Table A2: Plausible values for prevalence of mental health service use in Croydon, Lambeth, Lewisham and Southwark

	Percentage of unlinked records assumed to be missed matches by category of missingness among date of birth and postcode		
	Missing date of birth and postcode (A)	Missing date of birth but at least one postcode recorded (B)	Non-missing date of birth and at least one postcode recorded (C)
Lower limit	10%	10%	10%
Base case	50%	35%	25%
Upper limit	90%	60%	40%

$$Lower\ Limit = \frac{0.1 * (\#\ of\ unlinked\ records\ in\ A) + 0.1 * (\#\ of\ unlinked\ records\ in\ B) + 0.1 * (\#\ of\ unlinked\ records\ in\ C) + \#\ of\ linked\ records}{\#\ of\ unlinked\ records + \#\ of\ linked\ records}$$

$$Base\ Case = \frac{0.5 * (\#\ of\ unlinked\ records\ in\ A) + 0.35 * (\#\ of\ unlinked\ records\ in\ B) + 0.25 * (\#\ of\ unlinked\ records\ in\ C) + \#\ of\ linked\ records}{\#\ of\ unlinked\ records + \#\ of\ linked\ records}$$

$$Upper\ Limit = \frac{0.9 * (\#\ of\ unlinked\ records\ in\ A) + 0.6 * (\#\ of\ unlinked\ records\ in\ B) + 0.4 * (\#\ of\ unlinked\ records\ in\ C) + \#\ of\ linked\ records}{\#\ of\ unlinked\ records + \#\ of\ linked\ records}$$

Table A3: Number of Cafcass person IDs that link to a BRCID, by match step

Step	Forename	Surname	DOB	Postcode*	Number of Cafcass person IDs that link a BRCID	Number excluded
1	Exact	Exact	Exact	Exact	1456	-
2	Soundex	Soundex	Exact	Exact	143	-
3	Exact		Exact	Exact	49	-
4		Exact	Exact	Exact	67	-
5	Exact	Exact		Exact	304	-
6	Exact	Exact	Exact		1010	-
7	Soundex	Soundex	Exact		148	-
8	Initial	Characters 1-3	Exact		39	4
		Total**			2843	-
		Total after exclusions			2840	-

* At least one Cafcass postcode matches at least one CRIS postcode
** Row totals will not add up to the total as one Cafcass person ID may match to two or more BRCIDs across the different matching steps.

Table A4: Modelling sociodemographic and case characteristics against match status among women involved in proceedings in Croydon, Lambeth, Lewisham and Southwark between April 2007 and March 2019: odds ratios with 95% confidence intervals (n = 3226)

Variable	Odds Ratio	95% Confidence Interval
**Age at index set of care proceedings**		
Under 25 years old (ref)		
25-34 years old	0.82	0.65 to 1.04
35 years old and over	0.69	0.54 to 0.88
Age unknown	0.08	0.06 to 0.11
**Ethnicity**		
White or White British (ref)		
Black or Black British	0.65	0.50 to 0.83
Other	0.59	0.43 to 0.81
Ethnicity unknown	0.73	0.59 to 0.91
**Number of sets of care proceedings recorded in Cafcass**		
One (ref)		
Two or more	1.23	1.00 to 1.51
**Year (April-March that index set of care proceedings began**	1.00	0.97 to 1.02
**IMD 2010 quintile associated with address at index set of proceedings**		
1 – most deprived (ref)		
2	1.00	0.82 to 1.22
3	0.73	0.56 to 0.96
4 or 5 – least deprived	0.51	0.34 to 0.78
Address unknown	0.43	0.34 to 0.55
**Had an infant child subject to proceedings**	1.42	1.18 to 1.71
**Had at least one child subject to a care, placement or special guardianship order (i.e. having PR curtailed or terminated)**	1.44	1.20 to 1.73

Table A5: Modelling sociodemographic and case characteristics against match status among women involved in proceedings in Croydon, Lambeth, Lewisham and Southwark between April 2010 and March 2019: odds ratios with 95% confidence intervals (n = 2380)

Variable	Odds Ratio	95% Confidence Interval
**Age at index set of care proceedings**		
Under 25 years old (ref)		
25-34 years old	0.64	0.48 to 0.85
35 years old and over	0.52	0.39 to 0.70
Age unknown	0.06	0.04 to 0.08
**Ethnicity**		
White or White British (ref)		
Black or Black British	0.59	0.44 to 0.80
Other	0.51	0.36 to 0.75
Ethnicity unknown	0.63	0.47 to 0.83
**Number of sets of care proceedings recorded in Cafcass**		
One (ref)		
Two or more	1.28	0.99 to 1.65
**Year (April-March that index set of care proceedings began**	0.98	0.93 to 1.03
**IMD 2010 quintile associated with address at index set of proceedings**		
1 – most deprived (ref)		
2	0.93	0.74 to 1.17
3	0.68	0.49 to 0.93
4 or 5 – least deprived	0.46	0.28 to 0.74
Address unknown	0.40	0.3 to 0.53
**Had an infant child subject to proceedings**	1.25	1.00 to 1.56
**Had at least one child subject to a care, placement or special guardianship order (i.e. having PR curtailed or terminated)**	1.39	1.13 to 1.71

Cafcass data processing and cleaning

Cafcass data pre-processing included checking and validating gender, forenames and postcode.

- Person name fields were cleaned to remove any information which was not a name (e.g. Mr, Mrs, job title or role in the case) and only name was allowed, though hyphenated names were ok.
- Gender was validated against twenty years of the forenames of baby boys and girls provided by the ONS (Office for National Statistics, 2017). Where at least 99% of children born with a particular forename were of the same gender, that gender was compared against gender recorded in Cafcass, raising a flag for manual review if found to be different. This approach was also used to infer gender for those with missing gender in Cafcass.
- Where the postcode field was empty, the other address fields were searched using a regular expression to extract postcode for the address field. All postcodes were then cleaned and validated against the UK format.
- De-duplication of the individuals was performed. This involved blocking individuals according to the Soundex code for their gender and forename. Comparisons were made between all individuals within blocks based on the Jaro-Winkler distances between forename, surname, and date of birth (Winkler, 1990). Match probabilities were calculated using an implementation EpiLink in R (Borg & Sariyar, 2019; Contiero et al., 2005).

Figure A1: Proportion of Cafcass person IDs who correctly linked to a SLaM service user record, before de-duplication, (n = 2840) who had missing date of birth or no recorded postcode in the Cafcass data

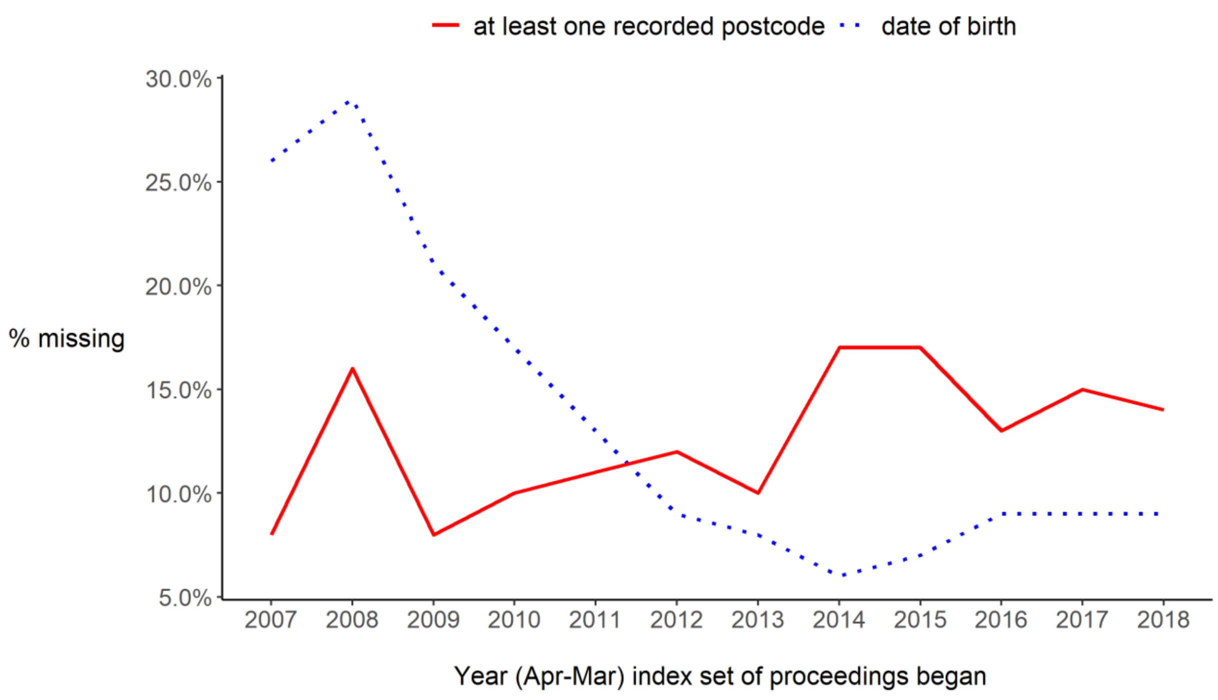

Table A6: Agreement in recorded ethnic group between Cafcass and CRIS among Cafcass person IDs that correctly link to a SLaM mental health service user record (n = 2840)

Ethnic group as recorded in Cafcass	Freq. with agreement in recorded Ethnic Group in CRIS	% with agreement in recorded Ethnic Group in CRIS
White or White British	762	86.49
Black or Black British	392	80.33
Asian or Asian British	28	66.67
Mixed Heritage	79	49.07
Other ethnic groups	11	26.19
Missing	131	10.69

Table A7: Ethnic groups recorded in CRIS where ethnic group is missing in Cafcass among Cafcass person IDs that correctly link to a SLaM mental health service user record (n = 2840)

Ethnic group as recorded in CRIS	Cafcass person IDs that link and are missing ethnic group in Cafcass (n = 1226)	
	Freq.	%
White or White British	551	44.94
Black or Black British	330	26.92
Missing	131	10.69
Inconclusive*	86	7.01
Mixed Heritage	66	5.38
Other ethnic groups	37	3.02
Asian or Asian British	25	2.04

* Ethnic group was non-missing in two or more CRIS electronic patient record systems and differed between them.

Table A8: Missingness among person identifiers in the CRIS database, by electronic patient record system

ePJS total service user records as at 8th July 2020: 356,814

	Count (%)				
	Forename	Surname	Sex	DOB	Postcode (at least one)
Not missing:	356,814 (100.00)	356,814 (100.00)	356,563 (99.93)	356,095 (99.80)	347,752 (97.46)
Missing:	0 (0.00)	0 (0.00)	251 (0.07)	719 (0.20)	9062 (2.54)

Iaptus total service user records as at 8th July 2020: 217,570

	Count (%)				
	Forename	Surname	Sex	DOB	Postcode (at least one)
Not missing:	217,570 (100.00)	217,569 (100.00)	216,905 (99.69)	217,546 (99.99)	216,891 (99.69)
Missing:	0 (0.00)	1 (0.00)	665 (0.31)	24 (0.01)	679 (0.31)

Appendix bibliography

Borg, A., & Sariyar, M. (2019). *CRAN - Package RecordLinkage*.

Contiero, P., Tittarelli, A., Tagliabue, G., Maghini, A., Fabiano, S., Crosignani, P., & Tessandori, R. (2005). The EpiLink record linkage software: presentation and results of linkage test on cancer registry files. *Methods of Information in Medicine*, *44*(1), 66–71.

Office for National Statistics. (2017). Baby names in England and Wales Statistical bulletins - Office for National Statistics.

Winkler, W. (1990). String Comparator Metrics and Enhanced Decision Rules in the Fellegi-Sunter Model of Record Linkage. In *Proceedings of the Section on Survey Research Methods*.
